# The impact of the COVID-19 outbreak on the connectedness of the BRICS’s term structure

**DOI:** 10.1057/s41599-022-01500-1

**Published:** 2023-01-03

**Authors:** Francisco Jareño, Ana Escribano, Zaghum Umar

**Affiliations:** 1 Faculty of Economic and Business Sciences, University of Castilla-La Mancha, Albacete, Spain; 2 College of Business, Zayed University, United Arab Emirates South Ural State University, Lenin Prospect 76, Chelyabinsk, 454080 Russian Federation

**Keywords:** Finance, Economics, Finance, Economics

## Abstract

This study aims to examine the impact of the different waves of the COVID-19 pandemic on the connectedness of the BRICS (Brazil, Russia, India, China, and South Africa) term structure of interest rates and its components (level, slope and curvature). For that purpose, this research applies the time-varying parameter vector autoregression (TVP-VAR) approach in order to assess the direction of spillovers among countries and factors and measure their contribution to the connectedness system. Our results show that the total connectedness measure changes over time, and the level and curvature components show connectedness that persists longer than the slope component, both in the first wave of the COVID-19 pandemic. Brazil and South Africa would appear as net transmitters of shocks, whereas China and India are net receivers. Finally, the most significant differences in the net dynamic connectedness between transmitters and receivers were focused on before and during the first wave of the COVID-19 pandemic crisis. Some additional impacts were observed during the last waves of the coronavirus pandemic. To our best knowledge, this is the first study on the connectedness between the yield curves of the BRICS economies and the COVID-19 crisis uncertainty according to the coronavirus MCI, by decomposing the yield curve into its factors (level, slope, and curvature).

## Introduction

In finance theory, one of the fundamental principles is the diversification of investments to reduce or mitigate the total risk of portfolios. This principle implies that investment portfolios should be composed of assets from different sectors and countries with weak or low correlations in order to try to eliminate or reduce portfolio risk as much as possible. In this sense, one of the roles of the BRICS countries (Brazil, Russia, India, China, and South Africa) in financial markets is the potential diversification and asset allocation benefit that the stock markets of these economies could bring to international portfolios (Bhar and Nikolova, 2009; Mensi et al., 2017b).

However, the phenomenon of globalisation has led to increased interlinkages, interdependence and interconnectedness of countries and global markets, which has been particularly noticeable during periods of economic crisis (Zaremba et al., 2019; Spierdijk and Umar, 2014), and especially damaging to emerging markets (Kenourgios et al., 2011; Syriopoulos et al., 2015). Moreover, this increase in interlinkages could lead to an increase in correlations between markets and economies, implying that the benefits of asset diversification could dissipate.

This fact has attracted the interest of researchers and academics, and in recent years, a growing number of studies have examined the interdependences and spillover effects surrounding past episodes of global economic turbulence (global financial crisis and European sovereign debt crisis) in the BRICS. The bulk of the literature has focused on spillover effects between the BRICS and developed markets, finding mixed evidence on the degree of dependence and spillover effects between countries (see, e.g., Aloui et al., 2011; Kenourgios et al., 2011; Dimitriou et al., 2013; Zhang et al., 2013; Syriopoulos et al., 2015; Bhuyan et al., 2016; Mensi et al., 2016, 2017a, 2017b; Bekaert and Harvey, 2017). Only a few studies investigate spillovers and dynamic effects among BRICS economies (see e.g., Kasman, 2009; Panda and Thiripalraju, 2018; Shi, 2021) and find that China and Russia play a dominant role in cross-market spillovers. These studies also highlight that BRICS economies, despite belonging to the same group of countries, have different market structures (Kasman, 2009), and are more independent from each other (Panda and Thiripalraju, 2018).

The ongoing COVID-19 crisis has dramatically affected all sectors and economies worldwide and represents an unprecedented global crisis episode with destructive economic damage never seen before, more harmful than the 2007–2008 global financial crisis (Goodell, 2020). The damaging effects of the pandemic are especially high in emerging countries, as these economies have weaker institutional and legal environments and higher levels of financial and social risks (Bretas and Alon, 2020; Hevia and Neumeyer, 2020). Among these countries, the BRICS represent the core of emerging countries, and the impact of the COVID-19 crisis on them is of particular interest because they account for a large share of the world’s gross domestic product and population (Bretas and Alon, 2020).

All these prior reasons favour the study of the interdependence of BRICS countries, and, in the current context, the COVID-19 pandemic crisis provides a unique scenario of structural breakdown to investigate the impact and spillovers among BRICS and the abrupt changes in spillovers, which is the motivation of this study, while adding to the knowledge and better understanding of the connectedness among BRICS countries. This research is also motivated by the demand from investors and financial market participants for assets with better returns and risk profiles during systemic crises such as the COVID-19 one. Although the bulk of the effects of the pandemic crisis on economic systems are yet to come, it is a fact that emerging markets will find it more difficult to overcome the crisis, as the response of these economies to the COVID-19 outbreak is limited across the board, not only at the health level, but also at the social, economic, and political levels.

Therefore, there are several reasons why the BRICS countries were selected for this study. First, the BRICS countries have experienced rapid economic growth over the past two decades but, individually, some of the countries exert very different dynamics, with China showing the highest growth rates, while the lowest ratios are those of South Africa. Secondly, such growth must be taken into account, as it presents important opportunities in the development trajectory of their financial markets. For instance, the stock markets of these economies are a potential source of diversification and provides asset allocation benefits for international portfolios. Third, globalisation, although it could have a negative effect on asset diversification benefits, is seen as having a positive effect on economic growth in developing countries and could serve as a growth differentiator for these countries. Fourth, BRICS countries are very important globally in terms of population, as half of the world’s population lives in these countries, and the size of their markets represents the majority of emerging economies. These countries are also implementing policies and creating agreements to build strong economic ties among themselves and also with other major industrialised nations of the world.

In this context, recent studies have focused on emerging markets to analyse the impact of the COVID-19 crisis in different settings and frameworks (e.g., Gubareva et al., 2020; Haroon and Rizvi, 2020a; Topcu and Serkan, 2020; Xu and Lien, 2021). While most of these studies focus on stock or foreign exchange markets, few papers concentrate on sovereign debt markets, from which the term structure of interest rates (TSIR) is extracted, which in turn can be used to analyse economic forecasting. The TSIR is a credible and reliable indicator of an economic downturn, and several authors have highlighted this role (e.g., Plakandaras et al., 2017a, 2017b; Plakandaras et al., 2019; Gupta et al., 2020a; Caldeira et al., 2020). Regarding the principal components of the TSIR (level, slope and curvature), according to Umar et al. (2018), among many others, any change in the level factor causes a parallel shift in the yield curve and is associated with the long-end of the yield curve. The slope factor can be seen as a short-term factor and an increase in it would amplify short-term rates more than long-term rates. The curvature factor can be interpreted as the medium-term factor, including flexibility to capture most of the varying shapes that the term structure of interest rates may exhibit. Thus, with this work we aim to fill this gap and investigate the connectedness between the yield curves of BRICS economies and uncertainty around the COVID-19 crisis. To the best of our knowledge, this effect has not been studied in previous empirical literature.

To investigate the BRICS bond market integration and the effect of the COVID-19 crisis on their interdependencies, we employ a two-step procedure. First, we decompose the yield curve into its Nelson and Siegel (1987) factors, level, slope, and curvature of each country. Second, we apply the time-varying parameter vector autoregression (TVP-VAR) methodology based on the dynamic connectedness framework of Diebold and Yilmaz (2009, 2012, 2014) proposed by Antonakakis and Gabauer (2017). This approach is useful in our context because bond market integration differs according to bond maturity and exhibits large co-movements in periods of turbulence with high uncertainty and high levels of volatility (Gabauer et al., 2020). Moreover, the TVP-VAR approach is appropriate for short data series, which is our case for a sample series around the outbreak of the COVID-19 crisis. This methodology was used by Antonakakis et al. (2018), Gabauer and Gupta (2018) and Antonakakis et al. (2020), Gabauer et al. (2020), Bouri et al. (2021), and Adekoya and Oliyide (2020), among others, and concluded that this approach overcomes the limitations of the Diebold and Yilmaz (2009, 2012, 2014) connectedness framework.

Briefly, we find interesting results in line with the previous literature, as the dynamic connectedness measure varies over time, being higher before and during the first wave of the pandemic. In addition, slight increases are observed in waves two and three of the coronavirus outbreak. On the other hand, the TSIR of Brazil and South Africa would appear as a net transmitter of shocks, while India and China would appear as net receivers. Finally, the main differences between countries were observed before and during the first wave of the pandemic, and these differences faded in the following waves of the COVID-19 pandemic, which have intensified again during the most recent waves.

This study is expected to provide new insights and contribute to the existing literature in several aspects. First, by exploring the role of BRICS countries and their interrelationships during the COVID-19 pandemic crisis. Second, in contrast to prior studies, this research incorporates the coronavirus MCI to analyse the connectedness of the BRICS region. Third, this study is unique in analysing the bond market integration of BRICS countries during the COVID-19 crisis and applying the TVP-VAR methodology to examine the bond market connectedness.

The remainder of this paper is organised as follows. Section “Literature review” presents the literature review. Section “Data and methodology” describes the study data and the methodology used in this research. Section “Results” shows the most relevant empirical results. Finally, section “Conclusions and implications” presents the most important concluding remarks.

## Literature review

In this section, we review the main relevant studies related with the topic of this work. We group the previous literature into three branches: first, studies related with the effects of the COVID-19 crisis on BRICS economies. Second, studies related with the connectedness in the BRICS countries. Third, studies examining the connectedness of the TSIRs.

### COVID-19 crisis on BRICS

Recent studies have focused on emerging markets to analyse the impact of the COVID-19 crisis in different contexts and frameworks (e.g., Gubareva, 2021; Haroon and Rizvi, 2020a; Topcu and Serkan, 2020; Janus, 2021; Xu and Lien, 2021; Zaremba et al., 2021; Bȩdowska-Sójkaa and Kliber, 2022; To et al., 2022). For example, Gubareva (2021) analyses bond market liquidity in emerging countries during the COVID-19 crisis and finds that liquidity has been severely damaged, with values far removed from pre-COVID-19 levels. Haroon and Rizvi (2020a) also examine liquidity in emerging markets and other aspects during the pandemic crisis. They find a negative relationship between the number of confirmed coronavirus cases and liquidity in financial markets. Other authors, such as Topcu and Serkan (2020), examine 26 emerging stock markets during the first wave of the COVID-19 outbreak and find evidence of large drops in all markets, especially in Asian markets, but all of them show slight improvements in mid-April. Xu and Lien (2021) investigate the effect of the COVID-19 crisis on the foreign exchanges interdependences of BRICS economies and find that the pandemic negatively affects the markets examined, particularly the dependences of the Chinese yuan and other BRICS currencies. Janus (2021) focuses on the impact of the first wave of the COVID-19 pandemic on the yield curve of a large number of emerging countries, including the BRICS countries, and find that the effects are highly divergent across economies. Zaremba et al. (2021) explore the impact of government interventions in the face of the COVID-19 crisis on some global stock markets, including some emerging countries such as the BRICS, and find that government policy responses decrease the volatility of local sovereign bonds significantly. In the same way, Zaremba et al. (2022) examine the impact of the COVID-19 pandemic on TSIRs of developed and emerging countries. Bȩdowska-Sójkaa and Kliber (2022) studies the impact of the COVID-19 pandemic restrictions on the sovereign risk of some Latin American and Asian countries, including some BRICS countries, by approximating the restrictions with the stringency index. They find that the more restrictions, the higher the volatility and the wider the sovereign bond spread. A recent study by To et al. (2022) also explores the impact of containment measures during the COVID-19 pandemic on different emerging and developed bond markets, including the BRICS, highlighting the importance of mass vaccination.

Another relevant issue is the role of media news in the COVID-19 crisis. Prior literature has pointed to the role of media news about infectious diseases on financial markets and investor sentiment (e.g., Barberis et al., 1998; Tetlock, 2007; Kaplanski and Levy, 2010; Groß-Klußmann and Hautsch, 2011; Su et al., 2017; González et al., 2021; Aharon et al., 2021; Umar et al., 2021d). In the current context of the COVID-19 outbreak, some authors have included variables able to capture the influence of media reporting in their analysis. Namely, Haroon and Rizvi (2020b), Cepoi (2020), Umar et al. (2021d, and 2022b) and Aharon et al. (2022) employ the coronavirus media coverage index (MCI), which measures the number of COVID-19-related news compared to other types of news, with the aim of exploring the impact of the pandemic on different financial markets.

### Connectedness on BRICS

As for the second branch of the literature review, the connectedness of BRICS economies has been studied, among others, by Bouri et al. (2018), Jiang et al. (2019), Dahir et al. (2020), McIver and Kang (2020), Hung (2021), Li et al. (2021), Umar et al. (2021e), Zhang et al. (2021), and Esparcia et al. (2022). Gabauer et al. (2020).

For instance, Bouri et al. (2018) investigate the potential interdependences between oil shocks (in terms of volatility) and government bonds for the BRICS, distinguishing between oil importers and exporters, and between positive and negative oil shocks, covering many possible scenarios in their analysis. In line with the previous paper, Umar et al. (2021e) explore the possible interdependences between decomposed oil shocks and stock markets for the Gulf Cooperation Council (GCC) and the BRICS, again focusing the findings on distinguishing the profiles of oil-importing and exporting countries. In contrast, Esparcia et al. (2022) explore the hedging role of gold during the pandemic by proposing short-, medium-, and long-term investment management strategies combining gold and stock market indices for the BRICS and G7 countries. The ex-post risk and return analysis revealed no significant differences between the combined strategies including indices based on BRICS and G7 countries as well as gold, unlike the study of dynamic correlations, where relevant differences emerge.

Another group of studies focuses on exploring potential spillovers between different financial markets in BRICS countries, such as Dahir et al. (2020), which investigates the dynamic connectedness between BRICS stock markets and Bitcoin. Similarly, Zhang et al. (2021) study dynamic volatility spillovers in G7 and BRICS stock markets by examining recent periods of economic turmoil, such as the ESDC, the China-US trade war, and the COVID-19 pandemic. As expected, volatility spillovers were amplified after these turbulent periods. Results along the same lines appear in McIver and Kang (2020), who also focus on different financial crises to shed light on the time-varying spillovers between BRICS and US equity markets. Jiang et al. (2019) also analyse BRICS equity markets but explore the connectedness with precious metals. They also find, as expected, that diversified portfolios can reduce risks. Finally, Ahmad et al. (2018) explore the return and volatility connectedness between the BRICS and three selected global sovereign bond markets of the US, the European Union (EU), and Japan. This research highlights the heterogeneity among sovereign fixed income securities issued by countries classified as BRICS.

On the other hand, Li et al. (2021) focus on geopolitical risks for the BRICS and explore potential dynamic spillovers with oil and gold prices, highlighting the relevant role of China in terms of net connectedness measures. Hung (2021) develops a similar analysis, as he studies the potential connectedness between BRICS stock markets and Economic Policy Uncertainty (EPU), highlighting, as expected, the increase in spillovers in times of crisis.

### TSIR connectedness

As for to the third group of papers analysing connectedness measures in BRICS countries, finally, regarding the connectedness of TSIRs, several authors have investigated the degree of bond market integration through TSIRs (Kumar and Okimoto, 2011) and by decomposing TSIRs into their three components (e.g., Jotikasthira et al., 2015; Ioannidis and Ka, 2018; Gabauer et al., 2020, Gupta et al., 2020a, b; Riaz et al., 2020; Aharon et al., 2022; Umar et al., 2022c, 2022d), and many of them have focused on analysing the connectedness between sovereign bonds and changes in oil prices (Filippidis et al., 2020; Nazlioglu et al., 2020; Umar et al., 2022a).

Thus, Ioannidis and Ka (2018) study the TSIR of some developed countries (half importers and half exporters) and their sensitivity to changes in crude oil prices. In addition, this paper decomposes the TSIR into the classical components of the yield curve, i.e., level, slope, and curvature. As expected, the results seem to depend on the source that caused the crude oil price fluctuation, as well as on the level of oil dependence of each country. Mensi et al. (2021) explore the dynamic connectedness between crude oil prices and European bonds, distinguishing between periods of expansion and crisis, such as the COVID-19 pandemic. In addition, they examine the portfolio design as a hedge combining crude oil futures and European bonds. The recent study developed by Urom et al. (2022) focuses on the evaluation of the time-varying integration between oil price shocks and interest rates in different economic areas, such as Asia, the US, and the EU. Similar studies, but focusing on Economic and Monetary Union countries, and leading oil producing and consuming countries, are found in Filippidis et al. (2020) and Umar et al. (2022a), respectively. The latter differs from the previous ones by breaking down high-frequency oil changes into risk, demand, and supply shocks, which can provide valuable information for market participants. Nazlioglu et al. (2020) explore some of the major oil exporting and importing countries and, in particular, return and volatility spillovers for their bond markets. They also concluded that it would be interesting to disentangle oil price changes into risk, demand, and supply shocks, as noted in the earlier work, Umar et al. (2022a), among others. Unlike all previous studies, the work of Shahzad et al. (2021) explores investment-grade corporate bonds (rather than government bonds) at different investment time horizons, also focusing on the interdependencies between crude oil prices and US corporate bond yields.

Following papers analysing the interrelationships between yield curves in different geographical areas, Umar et al. (2022c) explores potential interdependences between the level, slope, and curvature components of TSIRs for G7 countries at short-, medium- and long-term horizons. Their results would be crucial for portfolios managers, as the findings could provide insights into the interactions of TSIR fluctuations among G9 countries. In a similar analysis but focusing on the potential connectedness between the main components of the TSIR for the US yield curve and sector portfolios for the Islamic market, the study of Umar et al. (2022d) confirms that periods of economic crisis are associated with high levels of connectedness (in return and volatility), whose implications for investment portfolio design are beyond doubt. Aharon et al. (2021) also study the dynamic connectedness between the US TSIR (considering all three components), bitcoin and safe-haven currencies, highlighting the role of bitcoin as a safe-haven asset, especially in periods of economic turbulence. Another paper that examines time-varying spillovers between the different components of the G7 countries’ yield curves is that of Aharon et al. (2022), using the coronavirus MCI index. Interestingly, the methodology used allows distinguishing between risk-sending and risk-receiving countries in terms of connectedness measures, which also has relevant implications for portfolio managers.

Some other studies that are framed within the topic developed in this paper, and that are more related to the one we propose here are, for example, those of Ahmad et al. (2018), Janus (2021), and Umar et al. (2021f). On the one hand, Umar et al. (2021f) focus on analysing the return and volatility spillovers between the three main components of the TSIR and stock indices at the sector level. Unlike our proposal, this paper only analyses one country belonging to the BRICS group, namely China. Our research is also in line with that of Ahmad et al. (2018), but we extend the sample period to cover the analysis of the impact of the COVID-19 pandemic on the yield curves of the BRICS countries. Furthermore, this paper uses weekly frequency data, as opposed to the daily frequency we propose in our research. Finally, Janus (2021) focuses on the impact of the first wave of the COVID-19 pandemic on the yield curve for a vast number of emerging countries, among them, the BRICS countries. Unlike our paper that applies a connectedness method (TVP-VAR), Janus (2021) employs a Bayesian model.

To sum up, this work fills a gap in the literature by investigating the connectedness of the TSIR components of BRICS countries during the COVID-19-pandemic crisis. Building on Diebold and Li (2006) and Antonakakis and Gabauer (2017), the purpose of our research is to provide evidence on the connectedness between the yield curves of the BRICS economies and the uncertainty around the COVID-19 crisis as measured by the coronavirus MCI. Our dual procedure allows us to assess the direction of spillovers between countries and factors, and to measure their contribution to the connectedness system.

## Data and methodology

### Data

We collect daily data on zero-coupon sovereign yields to estimate yield curve factors for each country (Brazil, Russia, India, China, and South Africa). We employ monthly maturities and select 3, 6, 12, 24, 36, 48, 60, 72, 84, 96, 108, 120, 180, 240, and 360 months. Zero-coupon bond yields for our panel dataset of five countries were retrieved from the Bloomberg terminal. Our sample covers the period from 1 January 2020 to 28 February 2022 and hence includes the different six waves of the COVID-19 pandemic crisis.^1^

In addition, we collected daily media coverage data from RavenPack. We employ the coronavirus media coverage index (MCI) to measure the level of media coverage of COVID-19.^2^ The coronavirus MCI is calculated as the ratio between news sources covering the coronavirus over all remaining sources. The index is calculated on a daily frequency and ranges from 0 to 100 (a value of 50% means that half of the news sources on a day are currently covering news related to the COVID-19 crisis).

### Methodology

In this subsection, we present the two-step methodology employed in this study. First, we estimate the level, slope, and curvature of sovereign yield curves using the dynamic Nelson–Siegel model (Diebold & Li, 2006). Second, we employ the Antonakakis and Gabauer’s (2017) TVP-VAR connectedness approach.

#### Nelson and Siegel (1987) model in Diebold and Li (2006)

We apply Diebold and Li’s (2006) dynamic Nelson–Siegel model to estimate the yield curve of BRICS zero-coupon sovereign bonds. The dynamic model is a parsimonious model, which includes the three factors proposed by Nelson and Siegel (1987), obtained by decomposing the yield curve. These main components are Level (*L*_*t*_), which is related to the first principal component; Slope (*S*_*t*_), associated with the second principal component; and curvature (*C*_*t*_), related to the third principal component. The dynamic version of the Nelson–Siegel model with time-varying parameters is given by

1 $$y_t\left( \tau \right) = L_t + S_t\left( {\frac{{1 - e^{ - \lambda _t\tau }}}{{\lambda _t\tau }}} \right) + C_t\left( {\frac{{1 - e^{ - \lambda _t\tau }}}{{\lambda _t\tau }} - e^{ - \lambda _t\tau }} \right)$$

where *y*_*t*_(*τ*) denotes the yield of a zero-coupon bond with time-to-maturity *τ* at a particular point in time *t*, and the factor loadings of *L*_*t*_, *S*_*t*_, and *C*_*t*_ are the level, slope, and curvature of the yield curve, respectively. Parameter *λ*_*t*_ is the exponential decay rate of the exponential function. The loading on the level factor, *L*_*t*_ is equal to one for all maturities. The loading on the slope factor *S*_*t*_ is driven by the exponential function, starting at one, and decays monotonically to zero as the maturity increases. The loading on the curvature factor *C*_*t*_ starts at zero, takes positive values for medium-term maturities on a hump, and then decays to zero. Hence, *L*_*t*_, *S*_*t*_, and *C*_*t*_ can be interpreted as the long-, short-, and medium-term factors, respectively.^3^

#### The TVP-VAR methodology

To model the factor dynamics of the BRICS yield curves and their connectedness with the coronavirus MCI, we employ the Antonakakis and Gabauer’s (2017) time-varying parameter vector autoregression (TVP-VAR) methodology. The TVP-VAR method is an extension and improvement of the connectedness approach of Diebold and Yilmaz (2009, 2012, 2014), as it allows variances to vary via a stochastic volatility Kalman filter estimation with forgetting factors, allowing us to capture possible changes in the underlying structure of the data and because of the limitations related to the rolling window procedure of the Diebold and Yilmaz (2009, 2012, 2014) approach. This methodology has also been used, among others, by Antonakakis et al. (2018), Bouri et al. (2018), Gabauer and Gupta (2018), Antonakakis et al. (2020), Gabauer et al. (2020), Bouri et al. (2021), and Adekoya and Oliyide (2020), who concluded that this approach overcomes the limitations of the Diebold and Yilmaz (2009, 2012, 2014) connectedness framework.

We applied this methodology to the time series of the components of the yield curves of Brazil, Russia, India, China, and South Africa. We estimate the connectedness between the components of these economies and the coronavirus MCI to examine the extent to which the yield curves of these countries have been affected by the COVID-19 crisis.

The TVP-VAR model can be written as follows:

2 $$\begin{array}{*{20}{c}} {Y_t = \beta _tY_{t - 1} + {\it{\epsilon }}_t} & {\left. {{\it{\epsilon }}_t} \right|F_{t - 1}\sim N\left( {0,\,S_t} \right)} \end{array}$$

3 $$\begin{array}{*{20}{c}} {\beta _t = \beta _{t - 1} + v_t} & {\left. {v_t} \right|F_{t - 1}\sim N\left( {0,\,R_t} \right)} \end{array}$$

where *Y*_*t*_ and *Y*_*t*−1_ represent an *N* × 1 and an *N*_*p*_ × 1 dimensional vectors, respectively, *β*_*t*_ is an *N* × *N*_*p*_ dimensional time-varying coefficient matrix and *ϵ*_*t*_ is an *N* × 1 dimensional error disturbance vector with an *N* × *N* time-varying variance-covariance matrix, *S*_*t*_. The parameters *β*_*t*_ depend on their own values *β*_*t*−1_ and on an *N* × *Np* dimensional error matrix, *v*_*t*_, with and *Np* × *Np* variance-covariance matrix, *R*_*t*_.

The connectedness procedure of Diebold and Yilmaz (2014) rests on the generalised forecast error variance decompositions (GFEVDs) (Koop et al. 1996; Pesaran and Shin, 1998) and on the generalised impulse response functions (GIRFs). It requires that the TVP-VAR model should be transformed to its vector moving average (VMA) representation: $Y_t = \beta _tY_{t - 1} + {\it{\epsilon }}_t = \mathop {\sum}\nolimits_{i = 1}^p {\beta _{i,t}Y_{t - i} + {\it{\epsilon }}_t}$ . The GFEVD, $\widetilde \phi _{ij,t}^g\left( J \right)$, with a forecast horizon of *J*, was computed from the GIRFs $\psi _{ij,t}^g\left( J \right)$. The GIRFs represent the responses of all variables *j* due to a shock in variable *i*. Furthermore, the GFEVD, which can be interpreted as part of the variance that one variable *i* has over the others *j*, can be calculated as follows:

4 $$\widetilde \phi _{ij,t}^g\left( J \right) = \frac{{\mathop {\sum}\nolimits_{t = 1}^{J - 1} {\psi _{ij,t}^{2,g}\left( J \right)} }}{{\mathop {\sum}\nolimits_{j = 1}^N {\mathop {\sum}\nolimits_{t = 1}^{J - 1} {\psi _{ij,t}^{2,g}\left( J \right)} } }}$$

where $\mathop {\sum}\nolimits_{j = 1}^N {\widetilde \phi _{ij,t}^g\left( J \right) = 1}$ meaning that all variables together explain 100% of the forecast error variance of variable *i*; and $\mathop {\sum}\nolimits_{i,\,j = 1}^N {\widetilde \phi _{ij,t}^g\left( J \right) = N}$ meaning that all variables are included in the system.

From the GEFVD, the total connectedness index is constructed as follows:

5 $$C_t^g\left( J \right) = \frac{{\mathop {\sum}\nolimits_{i,t = 1,i \ne j}^N {\widetilde \phi _{ij,t}^g\left( J \right)} }}{{\mathop {\sum}\nolimits_{i,j = 1}^N {\widetilde \phi _{ij,t}^g\left( J \right)} }} \ast 100 = \frac{{\mathop {\sum}\nolimits_{i,t = 1,i \ne j}^N {\widetilde \phi _{ij,t}^g\left( J \right)} }}{N} \ast 100$$

This connectedness index allows us to estimate the degree to which a shock in one variable is transmitted to other variables, i.e., the degree to which a shock measured by the coronavirus MCI is transmitted to the yield curves of the BRICS economies.

From the total connectedness index, other connectedness measures that measure the direction of relationships can be derived. First, the transmission of a shock on variable *i*, i.e., on the coronavirus MCI, to all other variables ***j***, i.e., to the factors of the yield curves. This type of shock is called “*total directional connectedness to others*’ (TO) and can be calculated as follows:

6 $$C_{i \to j,t}^g\left( J \right) = \frac{{\mathop {\sum}\nolimits_{j = 1,\,i \ne j}^N {\widetilde \phi _{ji,\,t}^g\left( J \right)} }}{{\mathop {\sum}\nolimits_{j = 1}^N {\widetilde \phi _{ji,t}^g\left( J \right)} }} \ast 100$$

Second, the transmission of a shock on variable *i*, i.e., on the coronavirus MCI, which is received from all variables *j*, i.e., from the factors of the yield curves of the BRICS economies. This type of shock is called the “*total directional connectedness from others*” (FROM) and is defined as follows:

7 $$C_{i \leftarrow j,t}^g\left( J \right) = \frac{{\mathop {\sum}\nolimits_{j = 1,i \ne j}^N {\widetilde \phi _{ij,t}^g\left( J \right)} }}{{\mathop {\sum}\nolimits_{i = 1}^N {\widetilde \phi _{ij,t}^g\left( J \right)} }} \ast 100$$

Furthermore, it is possible to compute a net effect, i.e., the influence that variable *i* has on the network, the so-called “*net total directional connectedness*” (NET), by subtracting Eq. (7) from Eq. (6):

8 $$C_{i,t}^g = C_{i \to j,\,t}^g\left( J \right) - C_{i \leftarrow j,t}^g\left( J \right)$$

The values of Eq. (8) can be interpreted as follows: if $C_{i,t}^g \,>\, 0$, it indicates that variable *i*, i.e., the coronavirus MCI, influences the system more than it could be influenced by that. On the contrary, if $C_{i,t}^g \,<\, 0$, it means that variable *i*, i.e., the coronavirus MCI, is driven by the system. Finally, if $C_{i,t}^g = 0$, it indicates that variable *i*, i.e., the coronavirus MCI, does not have an influence, nor is it influenced by the system.

## Results

Table 1 presents the main descriptive statistics and unit-root tests for the time series of the three components extracted using the dynamic Nelson–Siegel model of Diebold and Li (2006). We observe that the mean values of the level coefficients, which reflect the long-term factor, are positive for all countries except China. The mean slope coefficients display negative values for all economies, except Russia, suggesting that yields increase with maturities. The mean curvature coefficients that represent the medium-term factors also present negative values for India and China, and positive values for the rest of the countries, and almost all are lower than the slope coefficients, suggesting that bonds with longer maturities are less liquid than those with medium-term maturities. We also found that the curvature coefficients are the most volatile of the three factors, which is in line with Gupta et al. (2020b). To test the stationarity of the time series, we transform the factor data series into their first differences. The standard unit root (Augmented Dickey-Fuller (ADF, 1979) and Phillips-Perron (PP, 1988)) and stationarity (Kwiatkowski-Phillips-Schmidt-Shin (KPSS, 1992)) tests confirm that all factor series are stationary.

**Table 1:** Descriptive statistics of the components of the BRICS term structure.

	Brazil	Russia	India	China	S. Africa
Panel A: Level					
Mean	0.0013	0.0106	0.0007	−0.0004	0.0029
Median	0.0003	0.0024	0.0014	0.0000	−0.0064
Maximum	0.3987	1.2205	0.3364	0.2093	1.3413
Minimum	−0.8095	−0.3436	−0.2534	−0.1920	−1.3961
Std. dev.	0.0919	0.0974	0.0458	0.0423	0.2202
Skewness	−1.1552	5.2489	0.0336	0.0610	0.5540
Kurtosis	16.1385	57.3351	12.5996	5.5958	13.3201
JB	4174.622^***^	71841.15^***^	2161.837^***^	158.4152^***^	2527.194^***^
ADF	−15.2409^***^	−20.4691^**^	−13.0004^***^	−17.0492^***^	−18.1776^***^
PP	−248.255^***^	−40.8125^***^	−58.9222^***^	−194.639^***^	−102.750^***^
KPSS	0.2842	0.2944	0.0068	0.0253	0.0256
Obs.	563	563	563	563	563
Panel B: Slope					
Mean	−0.0040	0.0068	−0.0030	−0.0003	−0.0067
Median	−0.0033	0.0015	−0.0018	0.0000	−0.0010
Maximum	0.9659	1.0961	0.2552	0.2718	2.1467
Minimum	−0.9369	−0.5362	−0.2699	−0.3303	−1.6668
Std. dev.	0.1310	0.0809	0.0526	0.0460	0.2566
Skewness	0.7284	4.8220	−0.1483	−0.0600	0.3599
Kurtosis	21.2486	72.5601	7.7312	11.0824	22.7363
JB	7861.682^***^	115687.3^***^	527.158^***^	1532.765^***^	9149.670^***^
ADF	−14.6934^***^	−12.4941^***^	−12.1265^***^	−17.3694^***^	−14.1025^***^
PP	−559.343^***^	−40.5587^***^	−119.226^***^	−136.746^***^	−84.4209^***^
KPSS	0.0048	0.2761^***^	0.0148	0.0370	0.0078
Obs.	563	563	563	563	563
Panel C: Curvature					
Mean	0.0050	0.0022	−0.0022	−0.0001	0.0036
Median	0.0040	0.0013	−0.0019	0.0000	0.0076
Maximum	1.3902	1.1250	0.8103	0.4781	2.1098
Minimum	−0.6200	−0.5505	−0.5716	−0.4646	−1.8392
Std. dev.	0.1922	0.1163	0.1021	0.0994	0.3324
Skewness	0.9508	1.5577	0.0757	0.1540	0.5856
Kurtosis	9.4148	19.4864	14.0085	6.3479	9.8875
JB	1050.131^***^	6603.688^***^	2843.380^***^	265.154^***^	1177.975^***^
ADF	−12.8762^***^	−14.6184^***^	−11.4316^***^	−16.4218^***^	−15.0614^***^
PP	−176.229^***^	−51.8080^***^	−140.222^***^	−193.014^***^	−169.418^***^
KPSS	0.2141	0.2544	0.0058	0.0489	0.0265
Obs.	563	563	563	563	563

This table shows descriptive statistics of the three main components of the daily BRICS term structure of interest rates. The sample period ranges from January 1, 2020 to February 28, 2022, including the waves of the COVID-19 pandemic. They include mean, median, minimum (Min.) and maximum (Max.) values, standard deviation (Std. Dev.) and Skewness and Kurtosis measures. JB denotes the statistic of the Jarque-Bera test for normality. The results of the augmented Dickey-Fuller (ADF, 1979) and Phillips-Perron (PP, 1988) unit-root tests, and the Kwiatkowski et al. (KPSS, 1992) stationarity test are also reported in the last three lines. As usual, ^*^, ^**^, ^***^ indicate statistical significance at the 10%, 5%, and 1% levels, respectively.

**Fig. 1:**
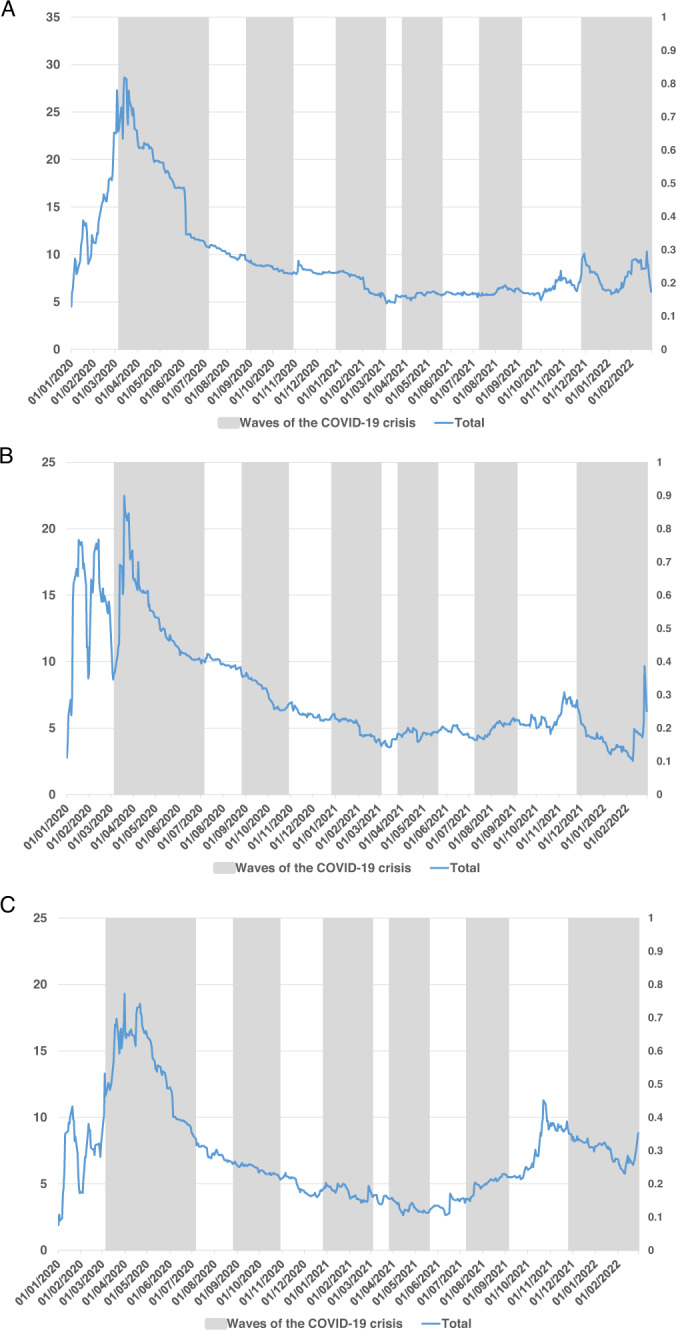
Dynamic total connectedness of the three components of BRICS term structure. Figure collects the time-varying total connectedness measure between the BRICS term structure of interest rates (decomposing into level (**A**), slope (**B**), and curvature (**C**)) using the TVP-VAR framework (Antonakakis and Gabauer, 2017), during the waves of the COVID-19 pandemic crisis.

**Fig. 2:**
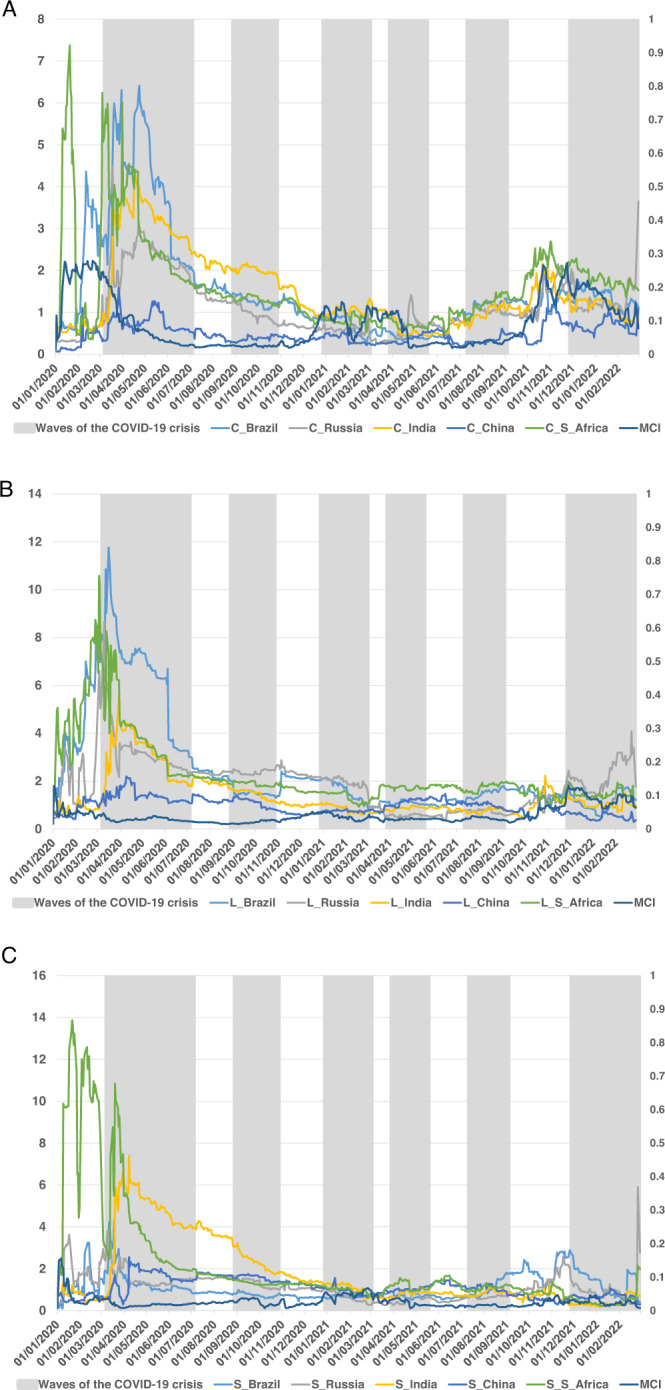
Dynamic contribution TO the system of the three components of BRICS term structure. Figure plots the time-varying connectedness TO measure between the BRICS term structure of interest rates (decomposing into level (**A**), slope (**B**), and curvature (**C**)) using the TVP-VAR framework (Antonakakis and Gabauer, 2017), during the waves of the COVID-19 pandemic crisis.

**Fig. 3:**
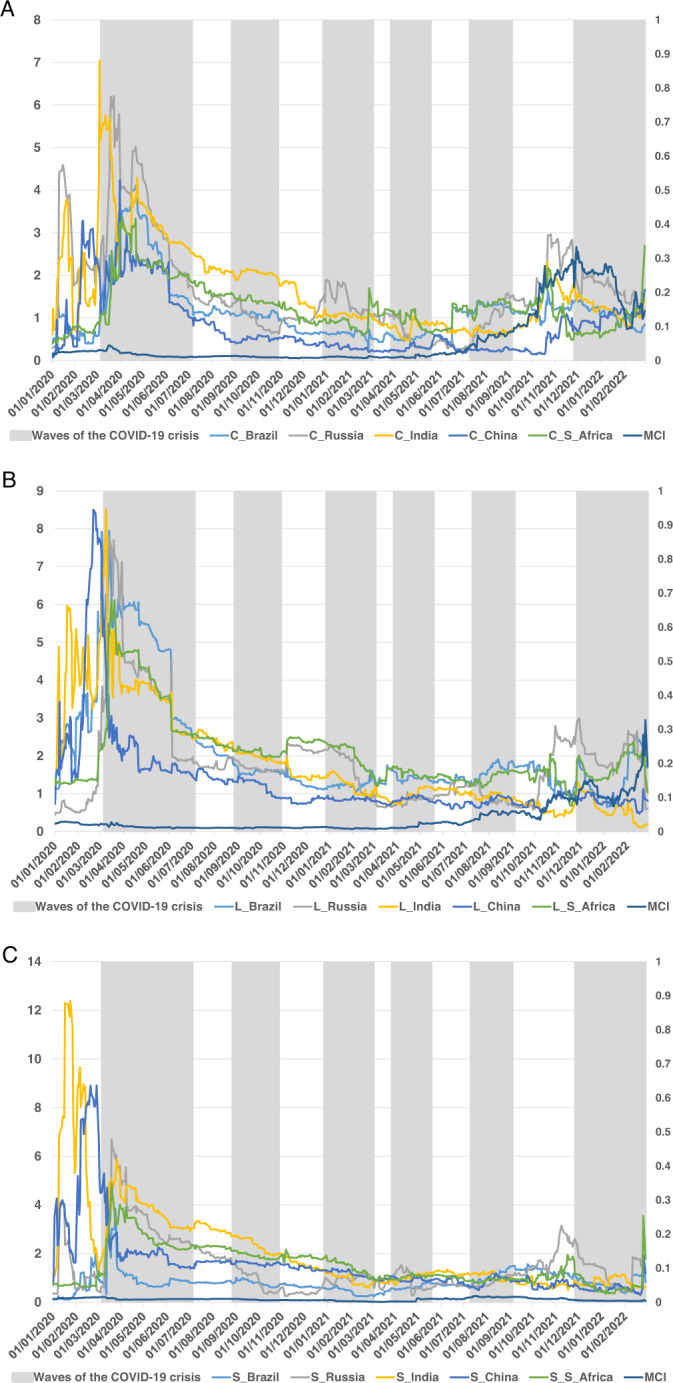
Dynamic contribution FROM the system of the three components of BRICS term structure. Figure collects the timevarying connectedness FROM measure between the BRICS term structure of interest rates (decomposing into level (**A**), slope (**B**), and curvature (**C**)) using the TVP-VAR framework (Antonakakis and Gabauer, 2017), during the waves of the COVID-19 pandemic crisis.

**Fig. 4:**
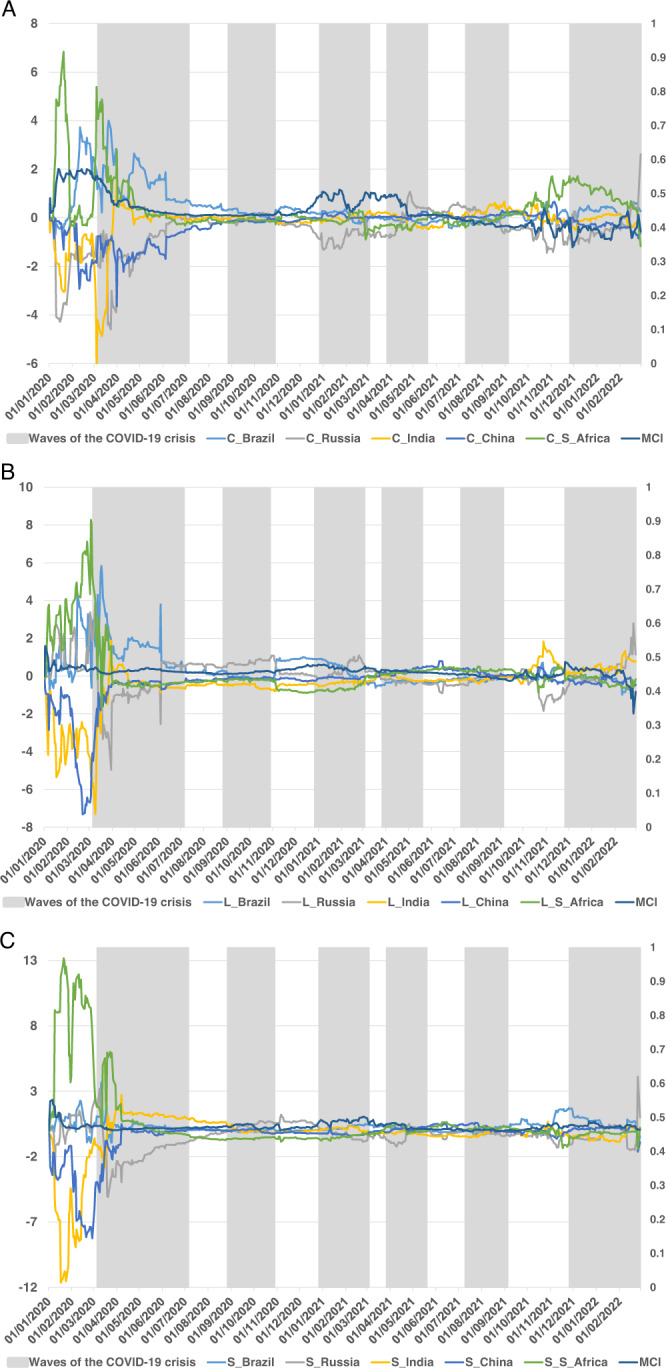
Net dynamic total connectedness of the three components of BRICS term structure. Figure plots the net time-varying connectedness measure between the BRICS term structure of interest rates (decomposing into level (**A**), slope (**B**), and curvature (**C**)) using the TVP-VAR framework (Antonakakis and Gabauer, 2017), during the waves of the COVID-19 pandemic crisis.

The results from the TVP-VAR connectedness approach are displayed in Figs. 1–4.

First, Fig. 1 shows the dynamic total connectedness of the BRICS term structure using the coronavirus MCI. As expected, dynamic total connectedness fluctuates over time, in line with Gabauer and Gupta (2018) and Umar et al. (2021b, 2021c, 2021d, and 2022b), among others. As observed in other financial markets, the highest levels of total dynamic connectedness in the three main components (level, slope, and curvature) occur during the first wave of the COVID-19 and, and, to a lesser extent, in the last waves of the pandemic. This result is line with the findings obtained by Janus (2021), as it seems that the most vulnerable countries found their situation worsen the most after the outbreak of the pandemic. Moreover, it is interesting to note that this increase is maintained for more days in the case of the level and curvature components, whereas in the case of the slope, the rise and subsequent fall occur very quickly. However, greater connectedness was observed for the slope component in the period leading up to the declaration of the global pandemic, heralding the impending health and economic crisis. This result points to the fact that the BRICS were prone to an abrupt increase in their short-term interest rates during the COVID-19 pandemic. Finally, we find no increased level of connectedness during the onset of the second and third waves of the global pandemic (both marked with shaded areas), only marginal spikes in the curvature component of the TSIR. These results are in line with those in Umar et al. (2021a). Finally, and fundamentally for the curvature component (related to the medium-term factors), we observed a significant increase in the level of connectedness at the end of the sample period studied, coinciding with the fifth and sixth waves of the pandemic.

Regarding the dynamic contribution TO the system of the three components of the BRICS term structure (Fig. 2), the term structure level shows an increasing trend during the first part of the sample, before the onset of the first wave of the COVID-19 crisis. Moreover, after the WHO declared the pandemic, the level of the term structure of interest rates showed a considerable increase in connectedness to the system, mainly in countries such as Brazil, South Africa, and Russia. However, this increase is smaller in India, showing a stable evolution in China. Thus, the dominant transmitters to the system are Brazil, South Africa, and Russia, mainly during the first wave of the COVID-19 pandemic. This result suggest that Brazil, South Africa and Russia are more likely to suffer a large increase in their long-term interest rates than India and China, in line with findings reached in Ahmad et al. (2018). Furthermore, in all cases, there is a decrease in connectedness TO before the end of the first wave of the pandemic, maintaining low and constant levels from that moment until the end of the fifth wave of the pandemic. Russia appears as the main net transmitter during the second wave, and South Africa during the third wave, although at much lower levels than during the first wave of the pandemic. In addition, the coronavirus MCI shows that the connectedness TO remains flat virtually throughout the entire sample period. It is interesting to note that Russia shows a connectedness TO the system that spikes at the end of the sample period, which coincides with the sixth wave of COVID-19 but could also be the prelude to Russia’s imminent attack on Ukraine. This level of connectedness increases considerably with respect to the rest of the countries in the level component of the term structure, suggesting an anticipation of a rise in long-term interest rates in Russia.

The slope of the term structure shows a slightly volatile connectedness to the system at the beginning of the sample period, highlighting the connectedness values reached in the case of South Africa, which shows a significant peak at the beginning of the first wave of the COVID-19 pandemic and descends rapidly before the beginning of April. When the connectedness shown by South Africa decreases, we observe a second important increase, which occurs in India. Russia also showed a peak (much less pronounced) at the beginning of the first wave of the pandemic, which fell in April. In all cases, the level of connectedness TO remained constant and low from April until the end of the sample period. In addition, India showed the highest connectedness level during the second wave of the pandemic, with a slight upturn in Brazil, during the last waves of COVID-19. Furthermore, in line with the level component, the slope and curvature component show a higher connectedness level at the end of the period analysed. This higher level of connectedness in the slope component of the yield curve could be heralding market uncertainty, not only because of the pandemic, but also because of Russia’s possible invasion of Ukraine.

The curvature of the term structure of interest rates shows a similar evolution, highlighting the levels of connectedness TO the system shown by South Africa. The most important peak occurred just before the start of the COVID-19 pandemic, with a drop in the connectedness measure occurring as early as March. In the case of the curvature component, South Africa, Brazil, and India show the highest levels of connectedness to the system during the first wave of the COVID-19 pandemic. These connectedness levels are reduced in all cases during the third and fourth waves, showing, in general, a decreasing trend until them. However, a slight increase in the connectedness measure of the curvature component of the TSIR should be noted in countries such as India and Russia during the fourth wave of the pandemic. Finally, an increase in the connectedness levels of the curvature component is observed in all countries, led by South Africa, just before the sixth wave of the COVID-19 pandemic, i.e., during the last months of the year 2021, with a final spike in Russia, which could be interpreted as a further foretaste of Russia’s imminent entry into Ukraine.

With respect to the total dynamic connectedness FROM the system (Fig. 3), we observe, in general, greater volatility in the level shown by the countries analysed. Specifically, in terms of the level component of the term structure of interest rates, we observe an increasing connectedness in the months prior to the first wave of the pandemic, which increases at the beginning of the wave, with the highest values from March to June 2020. Specifically, successive peaks were observed in India, China, Brazil, and Russia. From June 2020 onwards, there is a significant decrease in the level of connectedness in all cases, with lower and more stable values until the end of the second wave of the pandemic. Between the second and third waves, there was a slight increase in the connectedness measure in South Africa and Russia, which showed the highest levels of interdependence, as well as a slight rebound in Brazil in the middle of the third wave of the pandemic. Although China shows a high level of connectedness FROM at the beginning of the sample period, during the first wave of the pandemic, it appears as the country with the lowest level of connectedness FROM in the system. Russia, South Africa, and Brazil exhibit the highest values of the connectedness measure. It is interesting to note the rebound observed in the connectedness FROM for Russia, just before the sixth wave included in the study. Although to a lesser extent, other countries also show upturns, as is the case of South Africa, which appears recurrently as one of the countries most connected by international events.

As for the slope of the term structure, the FROM connectedness measure shows higher and more volatile values at the beginning of the sample period (January, February, and March 2020), reducing the level of this measure from that point until the end of the sample period. In this case, the initial levels shown by China and India stand out, as well as the peaks observed in Russia, India, and South Africa at the beginning of the first wave of the COVID-19 pandemic. However, Brazil showed the lowest level of connectedness. Finally, it is worth noting the peak observed at the beginning of the sixth wave of the pandemic in the case of Russia, which could be interpreted, again, as an early indicator of the situation triggered by the Russian invasion of Ukraine at the end of February 2022.

Finally, the connectedness measure shown by the curvature component exhibits slightly lower levels than the other components of the term structure of interest rates (in line with previous studies, such as Umar et al., 2018), although some higher levels are again observed at the beginning of the first wave of the COVID-19 pandemic, mainly in the case of India. However, milder peaks were also seen in Russia, South Africa, China, and Brazil. Furthermore, we observed small increases that coincided with the following (second and third) waves of the pandemic. In the curvature component of the term structure of interest rates, we observe a level of the connectedness FROM the system that increases in the last waves of the pandemic, mainly in South Africa (fifth wave) and Russia and India (sixth wave). In addition, the MCI index also experiences a strong increase at the beginning of the fifth wave, intensifying in the sixth wave of the COVID-19 pandemic.

Therefore, both connectedness measures (TO and FROM) confirm higher values in the first wave of the COVID-19 outbreak, that is, during the period characterised by a slowdown in economic activity all over the world, which would be indicative of a generalised fall in interest rates in the short, medium, and long-term. Moreover, the impact of successive waves is not as high as that of the first wave, although some effects can be observed in some components of the TSIR and for specific BRICS countries, mainly during the last wave of the pandemic, at the beginning of 2022.

Finally, Fig. 4 shows the net dynamic total connectedness as the difference between connectedness TO and connectedness FROM of the BRICS term structure of interest rates. First, in terms of the net connectedness measure of the level component of the term structure, the greatest differences between the BRICS connectedness levels are in the first part of the sample tested, that is, before and during the first wave of the COVID-19 pandemic. Specifically, Brazil and South Africa show a net transmitting position during the first part of the sample, although the latter goes on to show a net receiving position from April at the height of the first wave of the pandemic until the end of the sample period. China and India show a net receiving position throughout the sample period, while Russia shows an initial net receiving position that changes to virtual neutral position, becoming a net transmitter and reaching the highest values during the second wave of the pandemic. Finally, it should be noted that the differences between the net connectedness shown by the BRICS term structure of interest rates are considerably reduced during the first three quarters of the year 2021, increasing these differences just before and during the last wave of the pandemic. India’s net transmitting role in this last stage of analysis should be highlighted, as well as the net receiving profile shown by Russia just before the sixth wave of the pandemic, which becomes a net transmitter at the end of the period analysed, coinciding with Russia’s invasion of Ukraine.

As for the slope component, we again observe that the net connectedness measure shows the largest differences between countries from January to March 2020. These differences disappear from April to the end of the sample period. In the first part of the sample, South Africa shows clearly positive net connectedness, while China and India show negative net connectedness. Brazil and Russia held neutral positions. In the latter part of the sample period, which coincides with the Russian invasion of Ukraine, a net transmitter position is observed for Russia.

Finally, the curvature component of the term structure exhibits a similar path to the other components, showing the main differences from the beginning of the sample, i.e., before the onset of the first wave of the pandemic. At this time, the largest differences are observed between the net connectedness of South Africa and Brazil (positive), and that of India, Russia, and China (negative). From April onwards (including the second and third waves of the pandemic), the differences between the net connectedness values of the BRICS term structure are very low. Interestingly, the MCI would appear as a measure that allows separating the evolution shown by the net transmitter, neutral, and receiver positions. Coinciding with previous analyses, the net connectedness reactivates again at the end of the sample period, showing differences just before the last wave of COVID-19. South Africa and Brazil show net transmitter profiles again, while Russia exhibits a net receiver profile that switches to net transmitter during March 2022, coinciding with the outbreak of the Russia-Ukraine conflict. A much more in-depth study of this aspect would need to be addressed in further research.

## Conclusions and implications

This study is the first attempt to investigate the connectedness between the three components of the term structure of interest rates (level, slope, and curvature) of the BRICS countries (Brazil, Russia, India, China, and South Africa) and the RavenPack’s coronavirus Media Coverage Index (MCI), during the subsequent waves of the COVID-19 pandemic crisis by using the time-varying parameter vector autoregression (TVP-VAR) methodology. The period under study thus spans from January 1, 2020 to February 28, 2022, coinciding with the Russian invasion of Ukraine. The TVP-VAR approach allow us to estimate different dynamic connectedness measures and the research results indicate that there exists a high level of connectedness between the three components and the coronavirus MCI.

In particular, our empirical results confirm, as expected, that the total connectedness measure between the three components and the coronavirus MCI fluctuates over time, showing different magnitudes of change. It reaches its highest values during the first wave of the COVID-19 coronavirus crisis and, to a lesser extent, in the last wave of the pandemic. This result is indicative of the high levels of correlation between BRICS government bond markets, especially during the beginning of the crisis, and further supports the initial idea that diversification benefits diminish during times of crisis, in line with the results in Mensi et al. (2017b).

By quantifying the connectedness measures of the three components of the yield curves, we observe that the co-movements between Brazil, Russia, India, China and South Africa change during the pandemic crisis. We find that the level (in line with Gabauer et al., 2020) and curvature components of the term structure show levels of connectedness that perseveres longer than that of the slope component in the first wave of the COVID-19 pandemic and even during the sixth wave. This pattern is especially observed in the case of India and South Africa, suggesting that the government bond markets of these economies are more connected during the crisis and thus provide lower diversification benefits. The impact of the intermediate waves of the pandemic on the connectedness measures of the different components of the TSIRs of the BRICS countries is significantly lower than that observed in the first wave of the coronavirus pandemic. From this point of view, we observe how the interlinkages among the BRICS fluctuate during this turmoil period through the behaviour of the three components of their yield curves.

In addition, the term structures of Brazil and South Africa would appear as net transmitters of shocks, while China and India would be net receivers of shocks, keeping the Russian term structure in a neutral position. The most significant differences in net dynamic connectedness between transmitters and receivers are concentrated before and during the onset of the first wave of the COVID-19 pandemic crisis, although we also find a reactivation of these differences at the end of the sample period, i.e., during the sixth wave of the pandemic. These findings suggest that Brazil and South Africa are influential sources of spillovers and connectedness between BRICS markets. In the face of large spillovers, the remaining economies in the system are significantly affected by shocks from these countries. Therefore, these results would indicate that Brazilian and South African government debt would not be suitable for diversification benefits from investing in BRICS debt assets. Finally, this paper provides the first evidence of Russia’s net transmitter position in the system explored in this research, specifically, at the end of the sample period, coinciding with the onset of the Russia-Ukraine conflict between in late February.

All of these results have important implications for governments, policymakers, academics, and practitioners. First, our results are relevant for policymakers in the BRICS, who need to ensure that measures are taken to mitigate the effects of shocks. In addition, these results would have relevant implications for monetary policymakers because it is crucial to study possible short-, medium-, and long-term financial interdependencies between economic areas and potential contagion vulnerabilities. Finally, these results are also interesting for the implementation of diversification, asset allocation, and hedging investment strategies for international investment portfolios in BRICS assets, considering the insights gained from the findings of this study. These facts indicate the need for further in-depth research on these issues. Moreover, the uncertainty generated by the Russian invasion of Ukraine is also an issue that needs to be analysed in depth, as it represents a global uncertainty problem of difficult-to-quantify proportions.

## Data Availability

Data are available on request from the authors.

## Footnotes

1 The end of the sample period (28 February 2022) was marked by the absence of data from the Russian market after that date, mainly due to the war in Ukraine, the invasion of which took place on February 24, 2022.

2 RavenPack is one the leading data analytics providers in financial services, and provide insights generated automatically from real-time news provided from over 22,000 sources of news and social media. Source: https://www.ravenpack.com/.

3 Please refer to Diebold and Li (2006) for complete details of their methodology.
